# NLGN3 Upregulates Expression of ADAM10 to Promote the Cleavage of NLGN3 *via* Activating the LYN Pathway in Human Gliomas

**DOI:** 10.3389/fcell.2021.662763

**Published:** 2021-08-16

**Authors:** Ning-Ning Dang, Xiao-Bing Li, Mei Zhang, Chen Han, Xiao-Yong Fan, Shu-Hong Huang

**Affiliations:** ^1^Department of Dermatology, Shandong Provincial Hospital Affiliated to Shandong First Medical University, Jinan, China; ^2^Institute of Basic Medicine, Shandong Provincial Hospital Affiliated to Shandong First Medical University, Jinan, China; ^3^Department of General Surgery, The First Affiliated Hospital of Shandong First Medical University, Jinan, China; ^4^Department of Neurosurgery, The First Affiliated Hospital of Shandong First Medical University, Jinan, China

**Keywords:** NLGN3, ADAM10, glioma, LYN, proliferation

## Abstract

The neuron derived synaptic adhesion molecular neuroligin-3 (NLGN3) plays an important role in glioma growth. While the role of autocrine NLGN3 in glioma has not been well-studied. The expression of NLGN3 in glioma was detected using immunohistochemistry. We further explored its function and regulatory mechanism in U251 and U87 cells with high expression of NLGN3. Knockdown of endogenous NLGN3 significantly reduced the proliferation, migration, and invasion of glioma cells and down-regulated the activity of the PI3K-AKT, ERK1/2, and LYN signaling pathways. In comparison, overexpression of NLGN3 yielded opposite results. Our results further demonstrate that LYN functions as a feedback mechanism to promote NLGN3 cleavage. This feedback regulation was achieved by upregulating the ADAM10 sheddase responsible for NLGN3 cleavage. Inhibition of ADAM10 suppressed the proliferation, migration, and invasion of glioma cells; oppositely, the expression of ADAM10 was correlated with a higher likelihood of lower grade glioma (LGG) in the brain. Our study demonstrates that glioma-derived NLGN3 promotes glioma progression by upregulating activity of LYN and ADAM10, which in turn promote NLGN3 cleavage to form a positive feedback loop. This pathway may open a potential therapeutic window for the treatment of human glioma.

## Background

Malignant glioma is the most common type of brain tumors and is both highly invasive and lethal (Stupp and Roila, 2017). The median survival rate of glioblastoma multiforme (GBM) is 14.5–16.6 months (Bush et al., 2017; Uhm and Porter, 2017). The Neuroligins (NLs) are a family of cell adhesion molecules located in the postsynaptic membrane of neurons and mediated synapse formation (Cao et al., 2018). These play important roles in the formation of synaptic structures, neurotransmitters release, synaptic recognition, synaptic maturation, and signal transduction (Chubykin et al., 2005; Südhof, 2008; Aiga et al., 2011; Krueger et al., 2012). The Neuroligin family consists of NLGN1, NLGN2, NLGN3, NLGN4X, and NLGN4Y (Ylisaukkooja et al., 2005). NLGN3 is involved in the regulation of synaptic structure and function and plays an important role in the formation and remodeling of synapses in the central nervous system (Davey et al., 2010; Li et al., 2018). In recent studies, researchers found that neuronal activity promotes high-grade glioma (HGG) growth through modulating Neuroligin-3 secretion. Blocking gliomas’ acquisition of neuron-derived NLGN3 can inhibit cancer growth that is regulated by neuronal activity (Venkatesh et al., 2015). In the absence of NLGN3, or with the use of drugs to interfere with NLGN3, human HGG is inhibited in mice (Venkatesh et al., 2017). However, current research is limited to neuron-derived NLGN3 in the brain tumor microenvironment; whether NLGN3 secreted by tumor tissue itself can affect the growth of glioma cells remains unknown. We determined that NLGN3 is highly expressed by glioma tissue itself, but NLGN3’s role and the mechanism involved in glioma progression have not been reported.

LYN is a member of Src family kinases (SFKs). Src family kinases are widely expressed in organs and tissues. They can catalyze phosphorylation of tyrosine phosphorylation sites by binding to substrate proteins, thereby activating various nuclear factors to initiate transcription, regulating various intracellular biological processes. Studies have found that LYN is up-regulated involved in the occurrence and development of many tumors, such as colon cancer, breast cancer, and pancreatic cancer.

In the present research, we elucidated the functional effects of glioma-derived NLGN3 and discussed the feedback regulation pathway of LYN and NLGN3 in glioma cells to further improve our understanding of glioma progression.

## Materials and Methods

### Immunohistochemistry

A tissue microarray containing 38 glioma tissues and three normal tissues was purchased from Outdo Biotech (Shanghai, China). Immunohistochemistry (IHC) was performed to detect the expression of NLGN3 according to the method previously described (Barnes et al., 1996). Five random visual fields of each sample were selected and analyzed using Image-Pro Plus 6.0 software. The results were scored according to the proportion of positive cells: 1 (∼1–25%), 2 (∼26–50%), 3 (∼51–85%), and 4 (>85%). One was considered negative and two through four were considered positive.

### Cell Culture and Transfection

Human glioblastoma cell lines HS683, A172, U373, U251, and U87 were purchased from the American Type Culture Collection (ATCC, United States), and cultured DMEM medium supplemented with 10% FBS was used for cell culturing at 37 °C in 5% CO_2_. Transfection was performed using Lipofectamine 2000 according to the manufacturer’s instructions (Invitrogen, CA, United States). NLGN3 specific siRNA (5′-GCAGACAAAGUGGGCUGUAAUTT-3′) was purchased from Ribobio Co., Ltd. (Guangzhou, China). Overexpression plasmid of NLGN3 (pcDNA3.1-NLGN3), ADAM10 (pcDNA3.1-ADAM10), and LYN (pcDNA3.1-LYN) were purchased from YouBio Co., Ltd., and verified using sequencing. The empty vector (pcDNA3.1) was used as the control. U251 cells were treated with LYN inhibitor bafetinib (15 μM), DMSO treantment was as control. U87 and U251 cells were treated with an ADAM10 inhibitor, GI254023X, at a concentration of 10 μM.

### Quantitative Real-Time RT-PCR

Total RNA was extracted using an Ultrapure RNA Kit (CWBio, Beijing, China) and reverse transcribed into cDNA using a HiFiScript cDNA Synthesis Kit (CWBio, Beijing, China). Quantitative Real-Time RT-PCR (qPCR) was performed using UltraSYBR Mixture (CWBio, Beijing, China). The ADAM10 primer (F: 5′-ATGGTGTTGCTGAGAGTGTT-3′; R: 5′-GACTGCTCTTTTGGCACGC-3′) was purchased from Ribobio Co., Ltd. (Guangzhou, China). The QPCR procedure consisted of 40 cycles of 95°C for 5 min, 95°C for 30 s, and 60 °C for 45 s. The 2^−ΔCt^ method was used to calculate the expression of ADAM10.

### Western Blot and Antibodies

After transfection or treatment for 24 h, culture mediums of U87 and U251 cells were collected, and the cell lysates were extracted using a RIPA buffer. For every sample, 20 μg of protein or 20 mL of culture medium was subjected to SDS-PAGE. Protein bands were then transferred to a nitrocellulose membrane (Schleicher & Schuell Inc., Keene, NH, United States). The membrane was blocked in 5% non-fat milk for 2 h and then incubated with primary antibodies at 4°C overnight followed by a secondary antibody for about 1 h at room temperature. Finally, the protein bands were developed using an ECL development system (Amersham, Arlington Heights, IL, United States) and quantified by ImageJ software. The primary antibodies against Bax (Cat^#^ 60267-1-Ig, 1:1,000), Bcl2 (Cat^#^ 12789-1-AP, 1:1,000), C-Caspase-3 (Cat^#^ 19677-1-AP, 1:10,000), C-Caspase-3 (Cat^#^ 60004-1-Ig, 1:10,000), Cyclin D1 (Cat^#^ 60186-1-Ig, 1:10,000), p-AKT (Cat^#^ 66444-1-Ig, 1:2,000), AKT (Cat^#^ 10176-2-AP, 1:5,000), ADAM10 (Cat^#^ 25900-1-AP, 1:2,000), Flag (Cat^#^ 20543-1-AP, 1:5,000), E-cad (Cat^#^ 60335-1-Ig, 1:5,000), N-cad (Cat^#^ 66219-1-Ig, 1:5,000), CDK4 (Cat^#^ 66950-1-Ig, 1:5,000), and GAPDH (Cat^#^ 60004-1-Ig, 1:10,000), as well as the secondary antibodies, were all purchased from ProteinTech (Rosemont, IL, United States). The primary antibodies against NLGN3 (Cat^#^ ab244265, 1:1,000), p-LYN (Cat^#^ ab33914, 1:2,000), LYN (Cat^#^ ab1890, 1:2,000), p-ERK1/2 (Cat^#^ ab223500, 1:5,000), and ERK1/2 (Cat^#^ ab17942, 1:5,000) were purchased from Abcam (Cambridge, United Kingdom).

### CCK-8 and Colony Formation Assays

Cell proliferation was measured *via* CCK-8 assay. Cells transfected or treated with ADAM10 inhibitors for 24 h were seeded into a 96-well plate at a concentration of 1,000 cells/well. 10 μL of CCK-8 reagent (Solarbio Science & Technology Co., Ltd., Beijing, China) was added, followed by incubation at 37°C for 1.5 h. The OD value was measured at a wavelength of 450 nm. Cell viability was measured every 24 h.

Colony formation assay was performed to detect cell proliferation. After 24 h of transfection, cells were inoculated into 6 cm dishes at a density of 500 cells/dish and cultured at 37°C for 2 weeks. Then, cells were fixed with 4% paraformaldehyde for 30 min and stained with 0.1% crystal violet for 30 min. The number of colonies were counted and analyzed. All experiments were repeated three times independently.

### Wound-Healing Assay

After 24 h of transfection, the bottom of the dish was scratched with a pipette tip. The scratched wound was photographed under a microscope after an additional 24 h. The area of wound healing was analyzed using ImageJ software. All experiments were repeated three times independently.

### Transwell for Cell Invasion Assay

100 μL of melted Matrigel (BD Company, United States) was added to the upper chamber of a 24-well plate, and 500 μL serum-free medium was added to the lower chamber. 1 × 10^5^ cells treated for 24 h were transferred to the upper chamber. After 24 h of culture, the cells were fixed with 4% paraformaldehyde for 30 min and then stained with 0.1% crystal violet for 20 min. Five visual fields were randomly selected under the microscope and photographed for counting and analysis.

### Flow Cytometry

After transfection, the apoptosis of U87 and U251 cells were detected according to the manufacturer’s instructions (Beijing 4A Biotech). 100 μL (about 1 × 10^6^) cells were maintained in 5 μL Annexin V/FITC at room temperature for 5 min.

10 μL PI was used for cell stained and flow cytometer (BD FACSC anto II, BD Biosciences, United States) was used for the apoptosis analysis.

### Statistical Analysis

All data in this study were expressed as the mean + standard deviation (SD). Statistical analysis of the data was performed using SPSS 18.0 and GraphPad Prism 7.0 software. IHC results were analyzed by *t*-test, and the remaining data were analyzed using the One-way ANOVA method. *p* < 0.05 was considered statistically significant.

## Results

### Glioma-Derived NLGN3 Is Upregulated in Glioma Tissues

**FIGURE 1:**
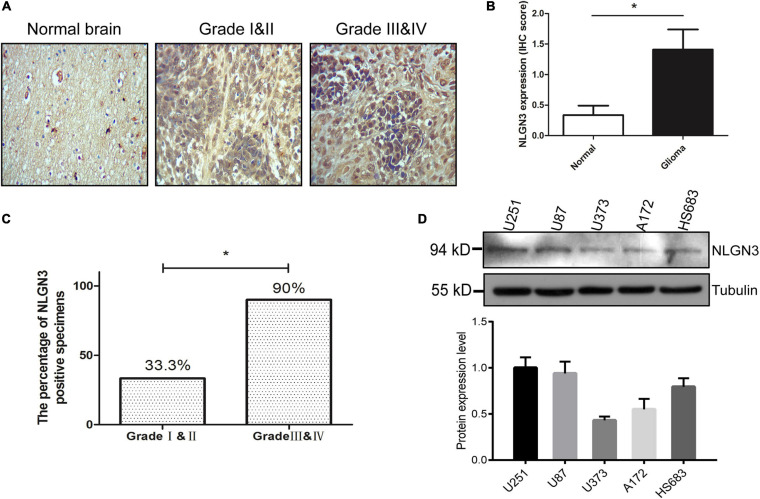
NLGN3 was overexpressed in human glioma. **(A)** The expression level of NLGN3 in human glioma was detected by IHC, and normal brain tissue was used as the control. The magnification of the image was 100x. **(B)** Statistical analysis of NLGN3 expression in glioma and normal tissues. **(C)** The percentage of NLGN3 positive specimens in high-grade glioma was significantly higher in comparison to low-grade glioma. **(D)** NLGN3 expression in five human glioma cell lines, U87, U251, HS683, A172, and U373, was detected using Western blot analysis. U251 and U87 cells showed higher levels of NLGN3 and were used for subsequent experiments. *p < 0.05.

First, we investigated the expression of NLGN3 in glioma by IHC detection of tissue microarray. As shown in Figures 1A,B NLGN3 was expressed in both normal brain and glioma samples, but the expression level of NLGN3 was significantly higher in glioma samples than normal brain tissues. We further divided the glioma samples into pathological I-II group and pathological III-IV group. The NLGN3-positive rate in the III-IV group was 90%, which was significantly higher than the 33.3% in the I-II group. This result suggests that glioma-derived NLGN3 is involved in glioma progression. Then, we detected the NLGN3 expression in five human glioma cell lines, U87, U251, HS683, A172, and U373, using Western blot (Figure 1D). NLGN3 was expressed in all five cell lines, but expression was highest in U251 and U87 cells, so these two cell lines were used for subsequent experiments.

### NLGN3 Promotes the Proliferation of U87 and U251 Cells

**FIGURE 2:**
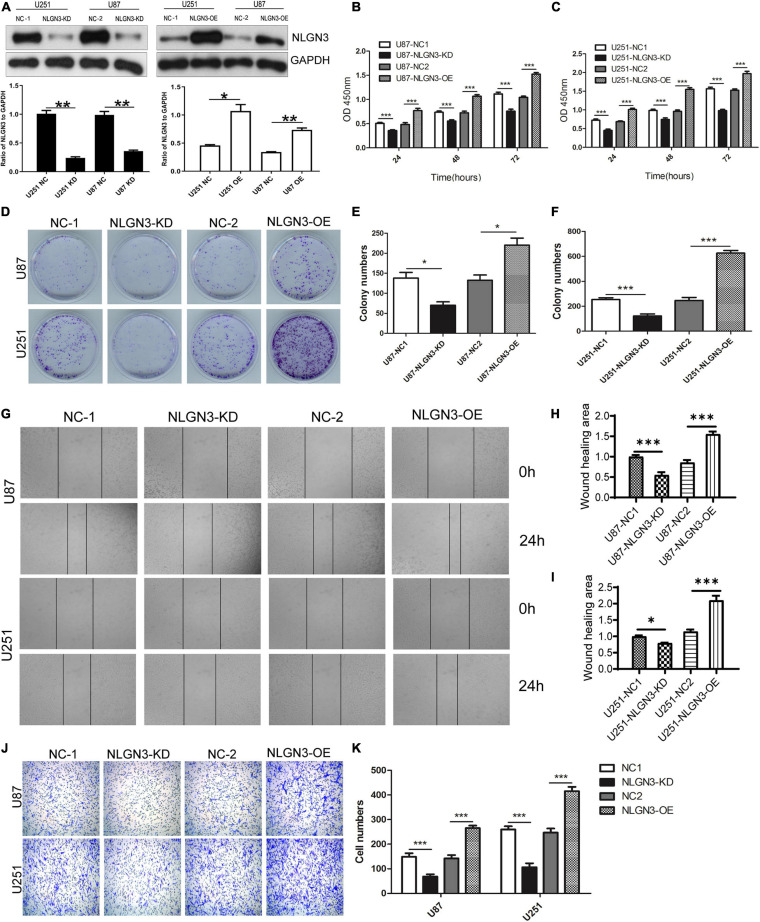
NLGN3 promoted the proliferation and metastasis of glioma cells. **(A)** NLGN3-specific siRNA and an overexpression plasmid was transferred into glioma cells to generate NLGN3 knockdown and overexpression cell lines, respectively. Western blot was used to detect NLGN3 expression. CCK-8 assay was performed to determine the proliferation of U87 **(B)** and U251 **(C)** cells. **(D)** Colony formation assay was performed to further confirm the proliferation of U87 **(E)** and U251 **(F)** cells. **(G)** The migration of U87 **(H)** and U251 **(I)** cells was detected using wound healing assay. **(J,K)** The invasion of glioma cells was determined using transwell assay. NC, negative control group; KD, NLGN3 knockdown group; OE, NLGN3 overexpression group; *p < 0.05; **p < 0.01; ***p < 0.001.

In order to investigate the role of NLGN3 in glioma, NLGN3-specific siRNA was transferred into the U87 and U251 cells to generate NLGN3 knockdown cells with negative-siRNA as control. Additionally, the NLGN3 overexpression plasmid was transferred into the U87 and U251 cells to generate NLGN3 overexpression cells with the empty vector pcDNA3.1 serving as control (Figure 2A). CCK-8 and colony formation assays were performed to determine the effect of NLGN3 on the proliferation of U87 and U251 cells. As shown in Figures 2B,C, knockdown of NLGN3 resulted in a significant decrease in cell viability compared to control cells; overexpression of NLGN3 demonstrated the opposite effect (*p* < 0.001). Moreover, the number of colonies in the NLGN3 knockdown group were significantly fewer than in the control group, with opposite results in the NLGN3 overexpression group in both U87 (*p* < 0.05) and U251 (*p* < 0.001) cells (Figures 2D–F). These results indicated that glioma-derived NLGN3 promoted the proliferation of human glioma cells.

### NLGN3 Promotes the Migration and Invasion of U87 and U251 Cells

Malignant glioma cells are highly metastatic and invasive, which severely limits treatment options and efficacy (Maher et al., 2001). To elucidate whether NLGN3 affects the metastatic potential of glioma cells, we further investigated the effects of NLGN3 on migration using wound healing and on invasion by transwell assay. As shown in Figures 2G–I, the NLGN3 knockdown group’s relative wound healing area decreased significantly in both U87 and U251 cells but increased markedly in the NLGN3 overexpression group compared with the control groups (*p* < 0.001). Transwell assay results showed that the number of invasive cells decreased significantly after knocking down NLGN3 and increased significantly after overexpression of NLGN3 (Figures 2J,K, *p* < 0.001). These results suggest that NLGN3 could effectively promote the metastasis of glioma cells.

### NLGN3 Inhibits the Apoptosis and Upregulates the Bcl-2/Bax Ratio I in Glioma Cells

**FIGURE 3:**
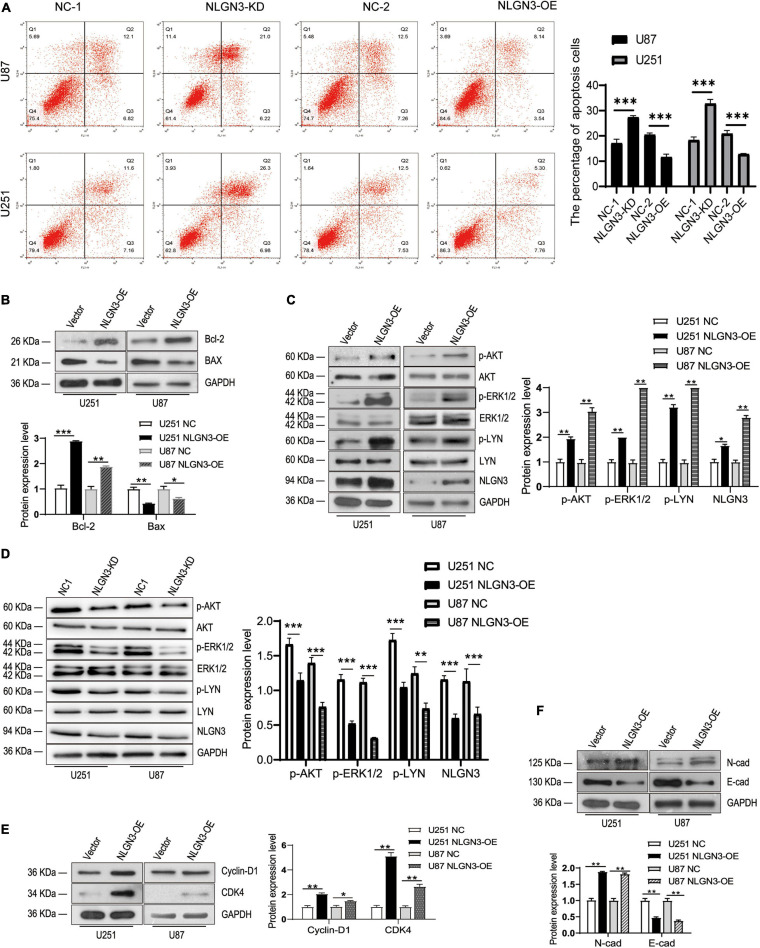
NLGN3 activated the AKT/ERK1/2 pathway and induces the EMT process in glioma cells. **(A)** The apoptosis was detected by flow cytometry in U87 and U251 cells. Then, the expression levels of NLGN3 downstream proteins were detected by Western blot. **(B)** NLGN3 upregulated Bcl-2 expression, and downregulated BAX expression. **(C,D)** NLGN3 upregulated the level of phosphorylation of AKT, ERK1/2, and LYN in U87 and U251 cells. **(E)** The expression levels of Cyclin-D1 and CDK4 increased in glioma cells with transfection of NLGN3 overexpression plasmid. **(F)** NLGN3 upregulated N-cad expression and downregulated E-cad expression. *p < 0.05; **p < 0.01; ***p < 0.001.

Then, we detected the apoptosis using flow cytometry in U87 and U251 cells. As shown in Figure 3A, the knockdown of NLGN3 significantly promoted the apoptosis of U87 and U251 cells, while the overexpression of NLGN3 significantly inhibited the apoptosis of U87 and U251 cells.

In the evolutionary conservative signaling pathway of apoptosis, the proportion of Bcl-2 family members is a key factor in apoptosis regulation; in particular, the Bcl-2/Bax ratio is the “molecular switch” that initiates apoptosis (Yang et al., 2017; Zhu et al., 2018). Our results showed that NLGN3 overexpression also promotes Bcl-2 expression while inhibiting Bax expression, which resulted in an increased Bcl-2/Bax ratio (Figure 3B).

### NLGN3 Activates the PI3K-AKT and ERK1/2 Pathway and Induces the EMT Process in Glioma Cells

To investigate the molecular mechanisms by which NLGN3 promotes glioma cell proliferation and metastasis, we examined the key signaling pathway for proliferation, the PI3K-AKT, ERK1/2, and LYN pathway, as well as the markers for proliferation, Cyclin D1 and CDK4, as well as the markers for EMT progress, N-cad and E-cad using Western blot. We found that NLGN3 overexpression significantly enhanced the phosphorylation levels of AKT, ERK1/2, and LYN compared to the control group in U87 and U251 cells (Figures 3C,D). Cyclin D1 and CDK4 are key proteins in the regulation of the cell cycle and are overexpressed in human gliomas (Yang et al., 2017; Sun et al., 2018). Our results showed that NLGN3 significantly promoted the expression levels of Cyclin D1 and CDK4 in U87 and U251 cells (Figure 3E). Compared with the control group, the expression of N-cad increased and E-cad decreased in the NLGN3 overexpression group, suggests that NLGN3 promotes the process of EMT (Figure 3F).

### Secreted NLGN3 Activates the LYN Signaling Pathway

**FIGURE 4:**
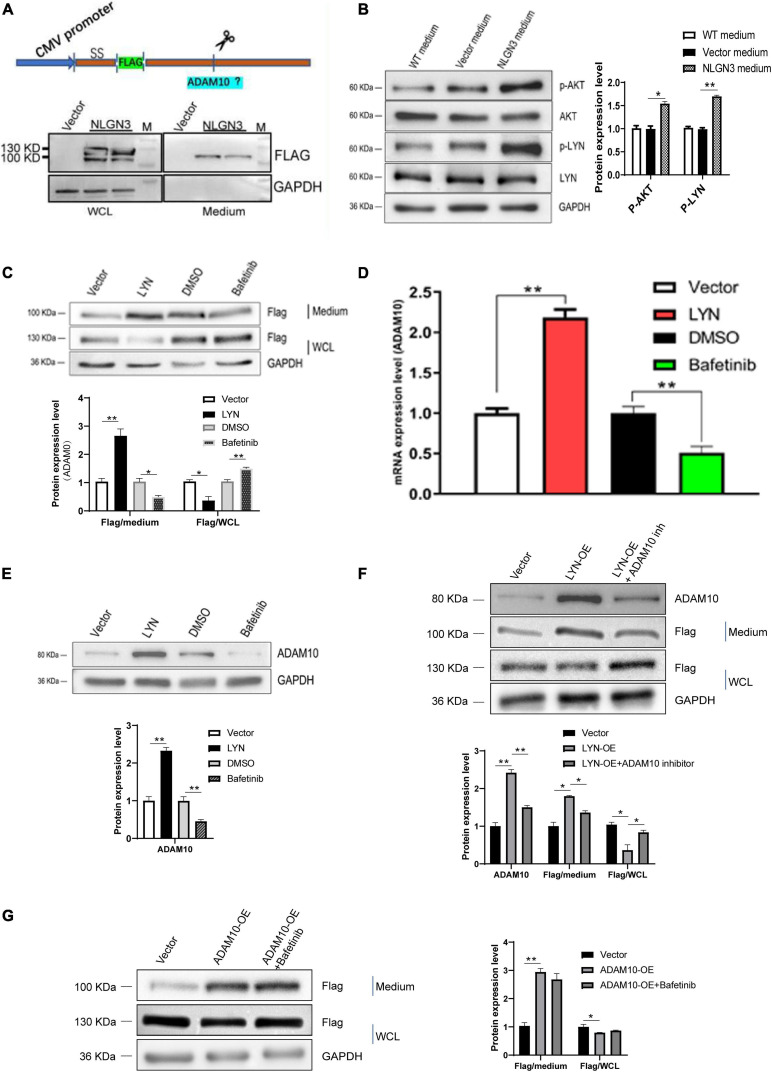
NLGN3 activated the LYN pathway to upregulate ADAM10, which in turn promoted cleavage of NLGN3. **(A)** The NLGN3 plasmid carrying the Flag fragment was transfected into U251 cells with an empty vector as the control. Western blot was performed to detect the Flag label. SS = signal sequence; M = maker. **(B)** The level of phosphorylation of AKT and LYN was detected after treatment with a NLGN3 plasmid, with an empty vector as the control. WT = wild type. **(C)** U251 cells were transfected with LYN or treated with bafetinib along with NLGN3 plasmid transfection, with an empty vector or DMSO as the control. Western blot was performed to detect the Flag label. WCL = whole cell lysates. **(D)** qPCR was performed to detect the expression of ADAM10 in U251 cells. **(E)** Western blot was performed to detect the expression of ADAM10 in U251 cells. ADAM10 expression was upregulated by LYN and inhibited by bafetinib. **(F)** Compared with the control cells, LYN overexpression increased the level of cleaved NLGN3, whereas decreased cleaved NLGN3 was observed after treatment with the ADAM10 inhibitor compared with LYN overexpression cells. **(G)** Overexpression of ADAM10 increased cleaved NLGN3 levels, but there was no significant change of cleaved NLGN3 levels after treatment with LYN inhibitors compared with ADAM10 overexpression group. *p < 0.05; **p < 0.01.

The activation of the LYN signaling pathway in glioma promotes the proliferation and metastasis of tumor cells (Dhruv et al., 2013, 2014). Therefore, we sought to elucidate whether NLGN3 is involved in the regulation of the LYN signaling pathway and through what specific mechanism. To investigate the proteolytic mechanism of NLGN3 secretion, we constructed a NLGN3 plasmid carrying the Flag tag in N-terminal. So both the full-length and cleaved NLGN3 (shedded NLGN3, active form) could be detected by Flag antibody. The plasmid was transfected into U251 cells, while an empty vector served as the control. Whole cell lysates (WCL) and cell culture medium were collected. Western blot analysis result shows that WCL contained both full-length and cleaved NLGN3 while the medium contained only cleaved NLGN3 (Figure 4A). Then, U251 cells were treated with the conditional medium (CM) of cells transfected with NLGN3 overexpression plasmid, whereas the culture medium of wild-type U251 or U251 cells transfected with empty plasmids were used as control. Western blot was performed to detect the phosphorylation levels of AKT and LYN in each group of cells. As shown in Figure 4B, secreted NLGN3 in the culture medium significantly upregulated the phosphorylation levels of LYN and AKT, indicating that secreted NLGN3 activated the LYN pathway in U251 cells. That is to say, the self-secreted NLGN3 was functional. So we used this plasmid to investigate the proteolytic mechanism of NLGN3 secretion. We next explored the role of the LYN pathway in NLGN3 shedding from U251 cells. As shown in Figures 4C,D, overexpression of LYN increased the level of secreted NLGN3, while the level of full-length NLGN3 decreased. Furthermore, after treatment with a LYN inhibiter, bafetinib (15 μM) the secreted NLGN3 level decreased while full-length NLGN3 increased. These results demonstrate that LYN promoted NLGN3 cleavage in U251 cells (Figures 4C,D).

### LYN Promoted the Release of NLGN3 Into Medium by Upregulating ADAM10 Expression

The results above indicate that there is a positive feedback regulation of the LYN pathway and NLGN3. What mediates the regulation of the LYN pathway to NLGN3 cleavage? Recent studies have shown that ADAM10 is responsible for NLGN3 shedding from glioma cells, so we further studied the role of ADAM10 in this positive feedback loop (Venkatesh et al., 2017). We detected the expression of ADAM10 using qPCR and Western blot analysis. As shown in Figures 4D,E, the mRNA and protein levels of ADAM10 increased in LYN overexpression cells compared with the empty vector group but decreased in bafetinib-treated cells compared with the DMSO group. This indicated that LYN promoted the expression of ADAM10 in U251 cells.

Next, to confirm the result, LYN overexpressed U251 cells were treated with ADAM10 inhibitor, GI254023X (10 μM). Compared with LYN overexpressed U251 cells, the ADAM10 inhibitor significantly reduced the level of cleaved NLGN3 in the medium but increased the level of NLGN3 in cells, suggesting that the ADAM10 inhibitor prevented the release of NLGN3 induced by LYN in U251 cells (Figure 4F).

We next transfected the ADAM10 overexpression plasmid into U251 cells to further verify its effect on NLGN3 cleavage. Results of Western blot analysis showed that the cleaved NLGN3 in the medium increased following overexpression of ADAM10, and the level of NLGN3 in cells decreased after the overexpression of ADAM10 (Figure 4G). Furthermore, the presence of LYN inhibitor, bafetinib (15 μM) in ADAM10 overexpression cells did not significantly change the expression level of NLGN3 both in medium and cells compared with ADAM10 overexpression group (Figure 4G). This data confirm that LYN activity play roles on the cleavage of NLGN3 at the upstream of ADAM10.

### ADAM10 Was Upregulated and Positively Correlated With NLGN3 and LYN Expression in Glioma, ADAM10 Inhibitor Suppressed the Migration and Invasion of Glioma Cells

**FIGURE 5:**
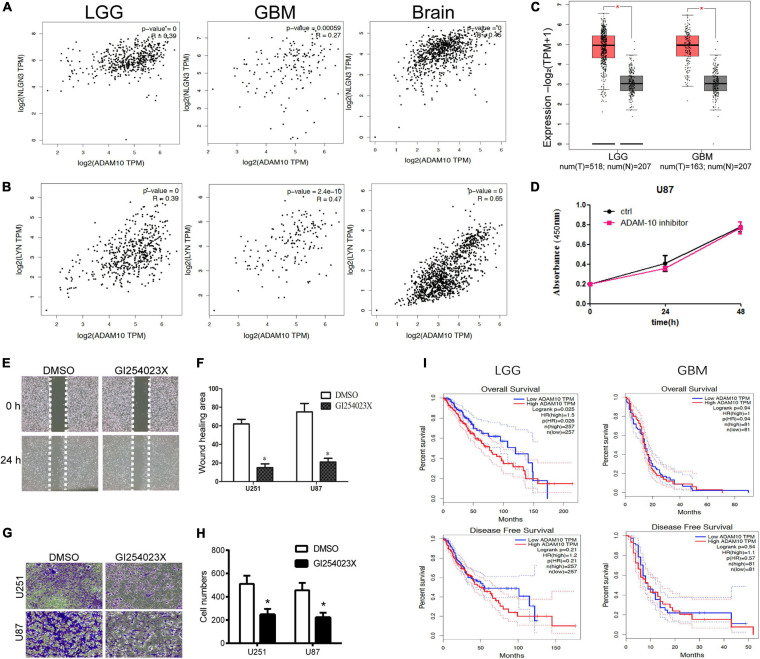
ADAM10 promoted the migration and invasion of glioma cells. **(A)** Correlation analysis between expression of ADAM10 and NLGN3 in glioma and normal brain tissue was performed using GEPIA. **(B)** Correlation analysis between expression of ADAM10 and LYN in glioma and normal brain tissue was performed using GEPIA. **(C)** The expression of ADAM10 in LGG and GBM was analyzed on GEPIA. **(D)** U87 cells were treated with ADAM10 inhibiter, GI254023X (10 μM), and CCK-8 assay was performed to detect the proliferation of each group of cells. **(E,F)** ADAM10 inhibiter, GI254023X, suppressed the migration of U87 cells. **(G,H)** GI254023X inhibited the invasion of U87 and U251 cells. **(I)** The relationship between ADAM10 and prognosis of patients with LGG and GBM was analyzed on GEPIA. *p < 0.05.

We analyzed the correlation between ADAM10, NLGN3, and LYN expression using the online GEPIA dataset (Tang et al., 2017). The expression of ADAM10 was positively correlated with NLGN3 in normal brain, LGG, and GBM tissues (Figure 5A) and showed a similar correlation pattern with LYN expression (Figure 5B). Expression analysis on GEPIA showed that ADAM10 was significantly upregulated in both LGG (*N* = 518) and GBM (*N* = 163) tissues compared to normal brain tissue (*N* = 207) (Figure 5C).

Then, we investigated the specific role of the ADAM10 inhibitor, GI254023X, in U87 cells. After treatment with the ADAM10 inhibitor, the OD values (450 nm) of U87 cells in the CCK-8 assay showed no significant differences compared to the control group (Figure 5D). We further investigated the role of the ADAM10 inhibitor in U87 and U251’s migration through wound healing and invasion through transwell assay. As shown in Figures 5E,F, GI254023X treatment led to a smaller wound healing area in U87 and U251 cells. The results of transwell assay showed that the GI254023X treatment reduced the invasive cell number compared with the control group in U87 and U251 cells (Figures 5G,H).

### High Expression of ADAM10 Predicted Poor Prognosis in LGG

The relationship between ADAM10 expression and the prognosis in LGG and GBM was analyzed *via* GEPIA. As shown in Figure 5I, LGG patients with lower ADAM10 expression showed significantly better overall survival. However, ADAM10 was not associated with *disease-free* survival in LGG patients. In comparison, ADAM10 was not associated with either overall survival or disease-free survival in GBM patients.

**FIGURE 6:**
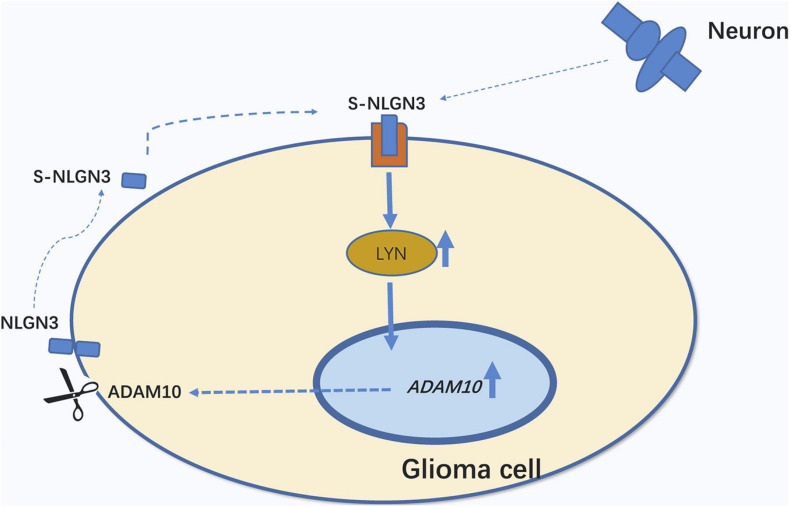
Schematic illustration of the positive feedback circle, s-NLGN3/LYN/ADAM10. Secreted NLGN3 derived from neurons and glioma cells activates LYN and then up-regulates ADAM10 expression, which can cleave NLGN3 to promote its secretion.

In summary, in the glioma microenvironment, NLGN3 secreted by neurons and glioma cells activates LYN and upregulates ADAM10 expression, which can cleave NLGN3 to promote its secretion (Figure 6).

## Discussion

In 2015, Humsa et al. revealed that neuronal activity promoted glioma growth *in vivo* (Venkatesh et al., 2015). Through the screening of activity-regulated secretion, they successfully identified the secreted NLGN3 as a leading protein that promotes the proliferation of glioma cells (Venkatesh et al., 2015). However, subsequent related research has been limited to neuron derived NLGN3, and the role of glioma-derived NLGN3 has not been reported. Using immunohistochemical analysis, we determined that NLGN3 was expressed abnormally highly in glioma tissues, suggesting that glioma-derived NLGN3 might be involved in glioma progression.

In our study, we first investigated the functions of glioma-derived NLGN3 in U251 and U87 cells by manipulating its expression. The results showed that NLGN3 significantly promoted cell proliferation, migration, and invasion in U251 and U87 cells. Previous studies suggest that neuron-derived NLGN3 plays an important oncogenic role in the progression of glioma, which is consistent with our study (Venkatesh et al., 2017; Liu et al., 2018).

Furthermore, we explored the underlying mechanism of NLGN3 in glioma cell lines. The AKT signaling pathways play a pivotal role in regulating cell proliferation, apoptosis, and cell movement (Altomare and Testa, 2005). Constitutive activation of these pathways often promotes tumor progression in many cancers (Altomare and Testa, 2005; Zhang et al., 2013). In this study, using Western blot, we found that p-AKT, p-LYN, and p-Erk1/2 were both upregulated by NLGN3 overexpression, which suggested that both AKT and Erk1/2 pathways were activated. In addition, the AKT signaling pathway also interacts with the mitochondrial-dependent apoptotic pathway, so we examined the effect of NLGN3 on apoptotic proteins (Mortenson et al., 2010; Zhao et al., 2015). The latest research shows that, NLGN3 is up-regulated in neuroblastoma tissues and increases the proliferation through activates the AKT pathway in neuroblastoma cells, which is consistent with our results (Li et al., 2019). NLGN3 resulted in upregulated Bcl-2 and downregulated Bax in glioma cells, which suggested that the mitochondrial-dependent apoptosis pathway was inhibited. The elevation of the Bax/Bcl-2 ratio induces cytochrome C to diffuse across the mitochondrial membrane into the cytoplasm, triggering cell apoptosis *via* caspases 9 and 3 (Lee et al., 2012). Cyclin D1 and CDK4 are downstream proteins of AKT which control cell cycle regulation and promote glioma cells proliferation (Chia et al., 2010; Gao et al., 2016; Kataria et al., 2016). We found that NLGN3 induced the expression of Cyclin D1 and CDK4 in U87 and U251 cells. This data suggested that NLGN3 promoted the occurrence and development of glioma. NLGN3 promoted N-cad expression and inhibited E-cad expression, indicating its involvement in the EMT process and its role in the development and metastasis of glioma.

In 2017, Humsa et al. reported that secreted NLGN3 from neurons can promote the progression of high grade glioma (HGG), and ADAM10 mediated cleavage led to the secretion of NLGN3 (Venkatesh et al., 2017). However, the mechanism of excessive secretion of NLGN3 remains unknown. In the present research, we found that secreted NLGN3 in cell culture medium promoted the level of LYN phosphorylation in glioma cells. LYN is a member of the Src family of tyrosine kinases and is mainly expressed in hematopoietic cells, nerves, liver, and adipose tissue (Xu et al., 2005). Several studies have shown that LYN activation can promote the progression of human glioma (Moncayo et al., 2018; Yang et al., 2018; Xavier et al., 2019). Furthermore, through the overexpression of LYN in U251 cells, we found that LYN promoted the expression of ADAM10, which in turn promoted the cleavage of NLGN3; LYN inhibitors could not rescue the effect of ADAM10. In addition, we found that the expression of ADAM10 in glioma and normal brain tissue was positively correlated with the levels of LYN and NLGN3. Therefore, we speculated that self-secreted NLGN3 enhanced ADAM10 levels by activating LYN, thereby promoting its own cleavage and forming a positive feedback loop.

Finally, we explored the effect of the ADAM10 inhibitor, GI254023X, on the function of glioma cells. The ADAM10 inhibitor suppressed the migration and invasion of U251 and U87 cells but did not affect cell proliferation. Previous studies have proven that GI254023X inhibits the proliferation of myeloma, breast cancer, stem/progenitor cells, and vascular smooth muscle cells (Hundhausen et al., 2003; Xiao et al., 2014; Chen et al., 2015; Mullooly et al., 2015). GI254023X inhibits metastatic ability but does not inhibit proliferation of glioma cells, suggesting that there may be a corresponding feedback inhibition pathway or other targets except ADAM10 in glioma cells. These hypotheses need to be confirmed by further studies. In addition, ADAM10 is associated with patient prognosis in LGG but not HGG, suggesting that ADAM10 has potential as a prognostic indicator for LGG. The different prognostic indications of ADAM10 in LGG and GBM suggest that the role of ADAM10 in LGG and GBM may be regulated by different pathways. In addition, the grade I-II and III-IV groups, LGG and HGG were involved in our study. In our research, LGG included grade I-II astrocytoma, hairy cell astrocytoma, pleomorphic xanthoastrocytoma, ganglioglioma, oligodendroglioma and mixed oligodendroglioma; HGG included astrocytoma, glioblastoma and malignant anaplastic astrocytoma (Wesseling and Capper, 2018). Due to the complexity of pathological classification and grading, the clinical value of NLGN3 in the diagnosis and treatment of gliomas needs to be verified in a richer sample size.

In conclusion, we proved that glioma-derived NLGN3 functions as a tumor promoter by upregulating AKT and Erk1/2 signaling pathways. We confirmed that secreted NLGN3 activates LYN and then promotes ADAM10 expression, which can cleave NLGN3 to promote its secretion. The NLGN3/LYN/ADAM10 axis promotes glioma progression *via* a positive feedback loop. This report will extend our understanding of the underlying molecular mechanisms of NLGN3 in gliomas, which may contribute to the development of novel diagnostic and therapeutic targets.

## Data Availability Statement

The original contributions presented in the study are included in the article/supplementary material, further inquiries can be directed to the corresponding author/s.

## Ethics Statement

This research study was approved by the Institutional Review Board of Shandong First Medical University.

## Author Contributions

N-ND and X-BL primarily performed the experiments, while N-ND analyzed the data and wrote the manuscript. X-YF and CH assisted with the experiments. MZ helped modify the manuscript. S-HH carried out the experiments design and manuscript drafting. All authors have edited and approved the final manuscript.

## Conflict of Interest

The authors declare that the research was conducted in the absence of any commercial or financial relationships that could be construed as a potential conflict of interest.

## Publisher’s Note

All claims expressed in this article are solely those of the authors and do not necessarily represent those of their affiliated organizations, or those of the publisher, the editors and the reviewers. Any product that may be evaluated in this article, or claim that may be made by its manufacturer, is not guaranteed or endorsed by the publisher.
